# The Operation of Canada’s Only Virtually Operated Radiation Oncology Service During the COVID-19 Pandemic

**DOI:** 10.1016/j.adro.2020.100634

**Published:** 2020-12-02

**Authors:** Claire Romani, Michael Conlon, Mike Oliver, Konrad Leszczynski, Michele Hunter, Kevin Lam, Silvana Spadafora, Andrew Pearce

**Affiliations:** a University of Toronto, Toronto, Ontario; b Health Sciences North, Sudbury, Ontario; c Northern Ontario School of Medicine, Sudbury, Ontario; d Sault Area Hospital, Sault Ste Marie, Ontario

## Abstract

**Purpose:**

Our institution operates a remote radiation oncology service in Northern Ontario, Canada. Since the start of the coronavirus disease 2019 pandemic, this center has operated without radiation oncologists on site owing to safety precautions, and this study seeks to understand the effect of this shift.

**Methods and Materials:**

Departmental level data reports were used to investigate differences in metrics between April to May of 2019 and April to May 2020. These metrics include the total number of referrals received, average wait time from referral to consult, the number of cases that underwent peer review before beginning treatment, the total number of fractions given over each period, patient-reported outcomes, and patient satisfaction. We also examined the importance of physical examinations and the use of SABR treatment.

**Results:**

There was an observed decrease in the number of referrals received, total number of fractions administered, and number of patients providing patient-reported outcomes. We observed no change in patient wait times, cases undergoing peer review before commencing treatment, or overall patient satisfaction. Challenges were identified in the collection of patient- reported outcomes and the conduction of physical examinations.

**Conclusions:**

This paper provides proof of concept that a radiation clinic can function entirely virtually in the short term without sacrificing patient satisfaction, efficiency, or safety.

## Introduction

Our institution has operated a remote radiation oncology service in Northern Ontario, Canada, since 2010. This program has on site radiation therapists, nurses, and 1 physicist but is operated remotely 3 days per week in terms of radiation oncologists since its inception, with radiation oncologists from the hub center traveling to the satellite center for in-person consults and follow-up visits on the remaining 2 days per week. The coronavirus disease 2019 (COVID-19) pandemic resulted in restrictions that prevented these radiation oncologists from working at multiple health care facilities. As a result, this satellite facility was operated entirely remotely from April until July of 2020. Although the pandemic has brought about changes in the daily operations of radiation clinics globally, with attempts to minimize the time patients spend in the hospital and interacting with staff,^1^ to our knowledge this is the only clinic operating entirely without the physical presence of a radiation oncologist in Canada. As such, this paper seeks to investigate the effect of this shift on numerous factors important to the operation of a radiation facility by comparing data from April and May of 2019 with the same months in 2020. These factors include the mode of visit (ie, telemedicine or in person), time from referral to consult, cases who had peer review conducted before the first radiation treatment, number of fractions of radiation given, number of patients seen, and patient satisfaction. Further, we will discuss how the use of SABR was continued during this period, and how other new challenges were overcome. This study will demonstrate the feasibility of operating a high-quality radiation treatment program entirely virtually for a limited period.

## Methods and Materials

This study examines the effect of the COVID-19 global pandemic on the operation of a satellite radiation oncology facility run completely virtually from April to May of 2020. Departmental level data reports from this period were compared with the same months in 2019 when the facility was operating normally These reports contained the necessary data to analyze total number of referrals, average wait time from referral to consult, the number of cases that underwent peer review before beginning treatment, the total number of fractions given over each period, as well as patient satisfaction with their radiation team.

Each referral was captured in the Mosaiq oncology information system (Elekta, Sweden). This allowed us to compare and determine whether fewer referrals were received during the start of the COVID-19 pandemic. Wait times from referral to consult were then calculated using the date of referral and the date of the patient’s first consult. Internal reports were used to determine the percentage of cases that underwent peer review before the commencement of treatment. The total number of courses of radiation administered was recorded in internal reports as was the number of fractions of radiation administered. Patient satisfaction data were available through internal surveys designed to provide physicians with feedback on the care being provided. Responses were anonymous with no patient identifiers were collected and were further anonymized to ensure the radiation oncologist could not be identified from the information included. We also describe qualitative changes, including the process for obtaining physical examinations and the operation of our SABR program in a remotely operating radiation treatment center.

Data were initially described graphically and with descriptives and frequencies. Before statistically testing, variables were assessed for normality, and subsequent metric comparisons between 2019 and 2020 were done using nonparametric tests (2-sample Wilcoxan rank-sum, Pearson χ^2^, Fisher exact) All testing was done using STATA, version 13.

## Results

**Figure 1:**
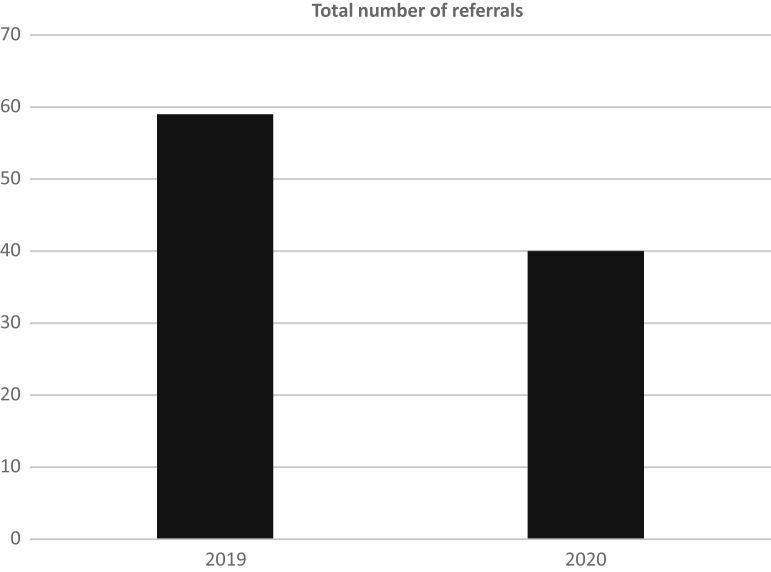
Total number of referrals between April to May 2019 and April to May 2020.

**Figure 2:**
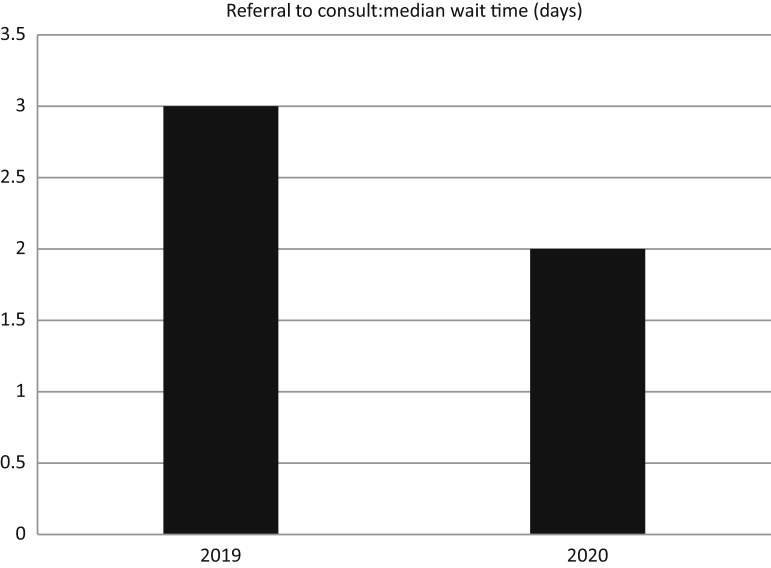
Median wait time from referral to consult.

In total, 40 referrals were received during April and May of 2020. This compares to 59 referrals in the same months of 2019, which is a reduction of approximately one-third (Fig 1). Despite the shift to all virtual appointments during April and May of 2020, referral to consult time in days remained similar to April and May of 2019, with a median of 2 days (range, 0-13 days) in 2020 compared with a median of 3 days (range, 0-34 days) in 2019 (z = 1.54, *P* = .12) (Fig 2).

**Figure 3:**
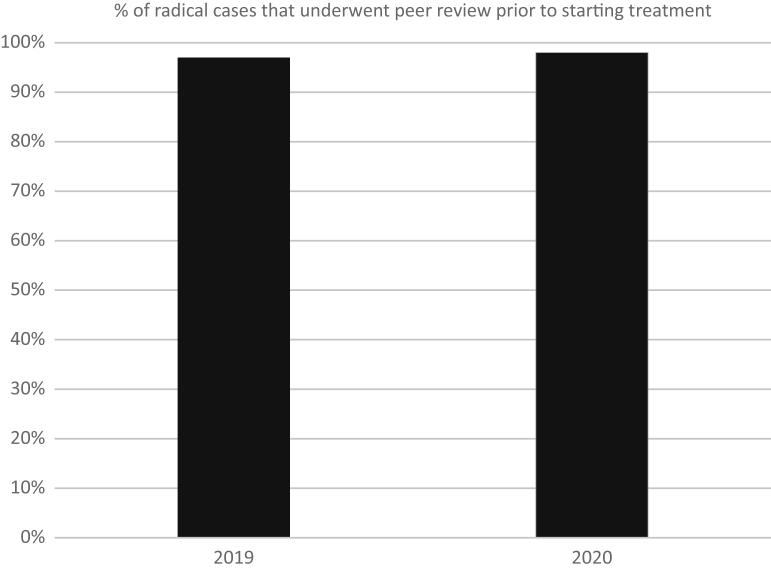
Percent of radical cases that underwent peer review before beginning treatment.

The number of patients whose cases underwent peer review before beginning radical radiation therapy remained high and increased slightly from the 2019 to the 2020 period. This value remained quite stable with a nonsignificant increase, rising from 97% in April to May of 2019 and 98% in April to May of 2020 (χ^2^[1, n = 220] = 0.16, *P* = .67; Fig 3).

The number of patient-reported outcome measures (PROMs) collected, evaluated through the Edmonton Symptom Assessment System (ESAS) tool, dropped substantially between April and May 2019 and 2020. The number of ESAS responses (each patient responds multiple times during a treatment course) over the 2019 period was 1661; however, only 10 responses were accrued over the 2020 period. The PROM scores evaluated by this tool cannot be compared.

**Figure 4:**
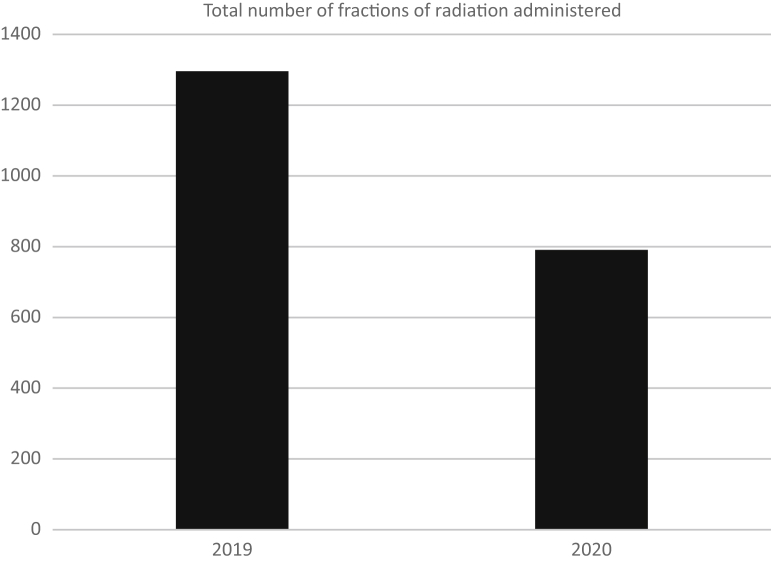
Total number of fractions of radiation administered.

There was a reduction of approximately 30% in the number of courses of radiation administered from the 2019 to the 2020 period, dropping from 129 down to 91 (Fig 4). There were fewer fractions of radiation administered per course in 2020 compared with 2019, but the difference was not significant (z = 1.46; *P* = .15). This additional change was produced by the adoption of multiple hypofractionation protocols across multiple tumor sites.

**Figure 5:**
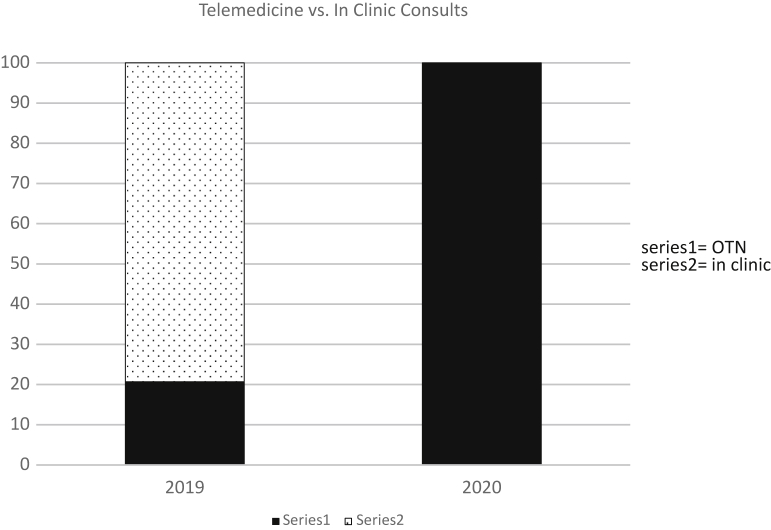
Percent of visits conducted via telemedicine.

With the radiation oncologists working entirely remotely, there was a significant increase in the number of visits conducted using telemedicine in 2020 versus 2019. In 2019, only 20.7% of visits were conducted through telemedicine, compared with 100% in 2020 since the outbreak of COVID-19 (Fisher’s exact test, *P* < .001) (Fig 5).

**Figure 6:**
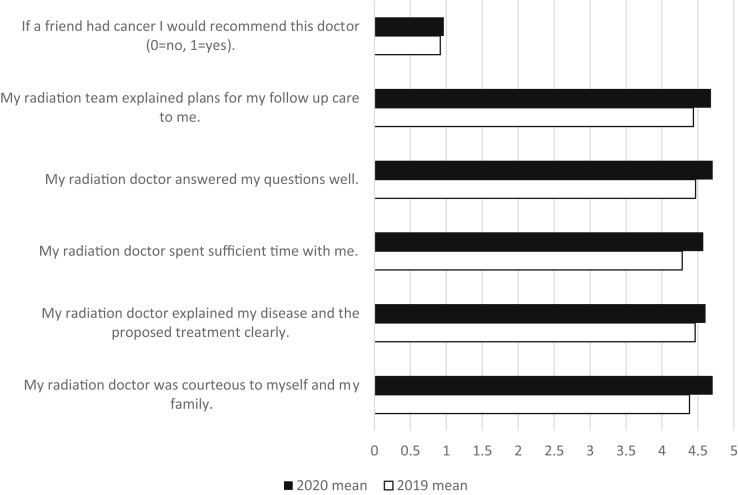
Patient satisfaction responses.

Patient satisfaction remained high during each period (Fig 6), and there were no statistically significant differences in patient response to any of the questions assessed on the department-administered patient satisfaction surveys that were returned by the subset of patients who completed the tool. Patients felt, and continue to feel, as though their radiation doctor was courteous to them and their families, their doctor spent sufficient time with them, and all their questions were answered well.

Our satellite facility has been performing SABR treatment since 2015. Because our facility was already operating virtually 3 days per week, a system was already in place for radiation oncologists and physicists to remotely visualize alignment images. Briefly, a remote-viewing system was built, consisting of a video capture card, virtual private network, and on-site viewing station and was designed to remotely and in real-time allow viewing of the Varian image guided radiation therapy workstation in our satellite facility. This system allows for physicists and radiation oncologists to review and assess the cone beam computed tomography matching process remotely, which is required for our SABR program to meet The American College of Radiology (ACR) and the American Society for Radiation Oncology (ASTRO) SABR guidelines.^2^ Initial requirements for SABR treatment included that the radiation oncologist attend the first SABR fraction (as a minimum) and the physicist attends all SABR treatment fractions. The remote-viewing station has permitted us to deliver high-quality SABR treatments without a radiation oncologist physically present at the distant cancer center, while still being able to meet the requirements for attending and directing the treatment process and for being readily available to address any issues arising during treatment delivery. Our program currently includes SABR for lung, liver, adrenal, nodal metastases, brain, and bony metastases. The remote-viewing system has provided the technology to instill confidence for the treating radiation therapists that the radiation oncologist can provide assistance and feedback for challenging cases. The presence of a radiation oncologist is very important in instilling confidence and maintaining treatment quality.^3^

With the patient work load decreasing due to the use of evidence based hypofractionated radiation treatment schedules and decreases in referrals, fewer staff were required to be onsite on a daily basis. Radiation oncologists, physicists, and radiation therapists (MRTs) were permitted and encouraged to work from home to the furthest extent possible to ensure that the limited and subspecialized staff providing radiation therapy services in Northeastern Ontario was as protected as possible from COVID-19. It was decided to split the MRTs into 2 separate teams rotating between working on site versus at home every 2 weeks. Those working at home had access to the electronic health record in the Mosaiq oncology information system, the Pinnacle radiation treatment planning system (Philips Health Care, Netherlands), and the hospital network system (business email system, electronic health record).

## Discussion

The COVID-19 pandemic has substantially affected the world, and radiation oncology is no exception. This pandemic has altered the way radiation departments have functioned in numerous jurisdictions. In Northeastern Ontario, the most evident and significant changes have been seen in our distant cancer center, where, owing to regulations preventing health care providers from working in multiple facilities, we were unable to continue our normal rotation of radiation oncologists and were instead forced to operate the facility without onsite radiation oncologists for over 2 months.

As a result of the pandemic, there was disruption in the health care system, limiting the ability of patients to be investigated for various clinical problems, some of which would inevitably be cancer. Many cancer screening services, including mammography and lung cancer screening, ceased.^4^ Additionally, many patients were hesitant to come into a hospital or doctor’s office to receive health care services. As a result, it was unsurprising that there was an observed drop in referrals of approximately one-third (Fig 1). As it is unlikely that the burden of cancer in Northeastern Ontario substantially changed over this period, we anticipate that unmet needs currently exist in our jurisdiction, which will likely need addressing in the coming months.

There was no significant increase in wait time from referral to consult despite the need to switch all consults to telemedicine in April and May of 2020, as can be seen in Figure 2. This is encouraging to see, as longer wait times would suggest potential additional burden on the patients who have already been dealing with a cancer diagnosis during a global health pandemic. Both the 2019 (4.96 days) and 2020 (4.67 days) averages fall well within the Ontario Health requirement of 85% of referrals being seen within 14 days.^5^ We interpret this result to mean that our staff was successful at rapidly implementing a change in the model of care delivery from a hybrid of in-person and virtual appointments to a completely virtual consultation process very quickly.

Although a reduction of 30% was seen in the total number of courses of radiation administered during April and May of 2020 compared with 2019, there was a larger observed reduction in the total number of fractions of radiation administered over this period, of approximately 40% (Fig 4). Significant effort was undertaken across Ontario to rapidly approve evidence-based hypofractionated fractionation schedules for multiple tumor types. This allowed us to care for a similar number of patients with cancer with fewer visits to the cancer center, smaller staff compliment, and reduced potential exposure to COVID-19 throughout the study period. These changes affected several tumor sites including single fractions for most palliation,^6^ 5-day adjuvant breast cancer treatments,^7^ 5-day neoadjuvant rectal cancer treatments,^8^ and 3-week radical brain tumor treatments.^9^

Because radiation oncologists were no longer physically present in our satellite center, in the 2020 period there were no in-person visits, with all consults and follow-up appointments occurring via telemedicine. Though there was an initial period of rebooking and rescheduling patients, this shift did not prove to be an insurmountable barrier and was facilitated as the technology for telemedicine visits was in place before the COVID-19 pandemic, with 20.7% of visits having taken place using telemedicine through April and May of 2019 (Fig 5). This technology ensured that all patients were still able to communicate directly with their radiation oncologist and allowed for visual examination of the patient by their treating physician.

Consistently high patient satisfaction scores across both periods are very encouraging (Fig 6). It demonstrates that although the radiation oncologists were meeting with patients only virtually, patients remained satisfied with the care they were receiving. Although the shift to all virtual radiation oncologist visits has been a large change and presented some challenges, it is extremely important that from the perspective of the patient they did not feel the care they received from their oncologist suffered.

An important change that occurred between April and May of 2019 and 2020 was the near absence of ESAS assessment responses in 2020. Because the completion of these surveys presently requires patients to use touch screens in kiosks located in the cancer center lobby, the concern of COVID-19 transmission using this high touch modality necessitated the discouragement of completing the assessments; however, an alternative system for ESAS completion was not put in place during this initial period due to the many other challenges that were being overcome at this time. Additionally, because the majority of virtual visits were conducted directly in the patients’ homes, no alternative system for the completion of these evaluations was rapidly deployed. This has unfortunately resulted in a lack of collection of PROMs, which are considered an important metric in cancer care,^10^ and the authors recognize the importance of restoring this functionality.

One important component of assessment for radiation treatment is physical examinations. With the pandemic preventing physicians from attending our distant cancer center, the oncologists have had to rely on designated community physicians to complete these examinations. We developed an internal mechanism to rerefer patients to specialist community physicians for the purpose of completing and documenting these necessary physical examinations. Results were then reported back to the oncologist. This has been done without major difficulties. The use of local physicians in conducting physical examinations, when warranted, has been functional as an emergency measure during the COVID-19 pandemic; however, it is likely not a long-term option. It is felt to be best practice for the treating radiation oncologist to personally perform a physical examination before the initiation of therapy, which is a standard that has now been reinstated.

This shift to remote operations has provided radiation therapists, physicists, and radiation oncologists the opportunity to work from home more frequently. Although there have been challenges associated with this remote system, such as the difficulty in obtaining physical examinations, the shift has not only provided patients with additional safety through reducing contact with hospital facilities but has also helped protect physicians and staff from COVID-19. The reduction of staff present in the cancer center improves the ability to physically distance and reduces the number of sources of exposure to the virus both for other staff members and for patients. Furthermore, physicians are not required to be in as many high-risk situations relating to COVID-19, related to transportation or lodging. Beyond the health benefits of working from home during a pandemic, working from home is reported by the physician group to increase job satisfaction, and some component of at-home work is planned to continue on a permanent basis. It has also decreased demand for clinic space in the cancer center and has the potential to decrease the need for office space as well. Components of these changes are likely permanent and will affect physical infrastructure needs on an ongoing basis.

The novel systems developed by our team have allowed SABR treatments to continue despite the fact that it typically requires a radiation oncologist to be present at the first treatment and a physicist on site for all treatments. Because of our remote viewing enhancements to the image guided radiation therapy image matching system, radiation oncologists and physicists are able to review and assess the alignment of computed tomography scans in real-time and provide immediate feedback if necessary. As such, the treatment team can continue to provide this treatment while adhering to the strict ACR-ASTRO guidelines for SABR treatment.^2^

Owing to the regular collection and storage of a broad range of performance metrics and patient data, we were able to directly compare a variety of metrics during and 1 year before the COIVD-19 global pandemic, allowing us to appropriately evaluate the effect on our operations during a period where we were required to operate completely virtually with respect to our radiation oncologists. We were able to compare metrics ranging from wait times and number of fractions administered, providing insight into the function of the center at a high level, to patient satisfaction at the individual level, providing us with a more holistic view of how the shift to a completely virtual facility affected operations.

One limitation of this study is that it only examined the early response to the pandemic. As the situation has continued to evolve through June and July, it is possible that the results seen here may differ. Additionally, because no alternative system for collecting ESAS data was put into place, resulting in an extreme drop in the number of patients who completed the assessment, we were not able to directly compare PROMs. As such, we cannot determine whether factors like patient fatigue, anxiety, pain, or overall wellbeing were affected as a result of COVID-19 or the changes introduced into the system.

## Conclusions

The COVID-19 pandemic has altered the way many radiation oncology facilities are functioning. To our knowledge, we report here the operation of the only completely virtual radiation oncology facility in Canada during the COVID-19 pandemic. In all, these results suggest that it is entirely feasible to operate a radiation clinic remotely for a short period of time during a state of emergency. Longer-term operation would require modifications to the operation to increase sustainability. Although these changes were born out of necessity during a worldwide pandemic, they provide a proof of concept that radiation therapy can be delivered in smaller centers closer to patients' homes with a heavy reliance on modern technology to maintain safety, efficiency, and patient satisfaction.
